# Prognostic value of the central venous-to-arterial carbon dioxide difference for postoperative complications in high-risk surgical patients

**DOI:** 10.1186/cc9458

**Published:** 2011-03-11

**Authors:** E Robin, E Futier, O Pires, G Lebuffe, B Vallet

**Affiliations:** 1 University Hospital of Lille, Université Nord de France, Lille, France; 2 University Hospital of Clermont-Ferrand, France

## Introduction

Tissue hypoperfusion is a key trigger of postoperative organ dysfunction. Our objective was to evaluate the prognostic value of the central venous-to-arterial carbon dioxide difference (PCO_2_) gap, a global index of tissue perfusion, in patients after major abdominal surgery.

## Methods

A prospective and observational study of 115 patients admitted to the ICU following major abdominal surgery. In all patients, measurements of the PCO_2 _gap, central venous oxygen saturation (ScvO_2_), serum lactate and conventional hemodynamic and biological parameters were performed on admission (H0), and over 6 hours until 12 hours after admission. Postoperative complications, the duration of mechanical ventilation, and the hospital length of stay and mortality up to 28 days were characterized using standard definitions. Area under the ROC curves for PCO_2 _gap, ScvO_2 _and lactate were calculated and compared to discriminate between patients with and without complications.

## Results

A total of 78 patients developed at least one complication including 57 (50%) patients with postoperative septic complications. At T0 there was no significant difference in demographic and hemodynamic data, type and duration of surgical procedures between patients with and without complications. There were nine deaths (7.8%). There was a significant difference for PCO_2 _gap (8.1 ± 3.2 mmHg vs. 5.5 ± 2.8 mmHg, *P *< 0.001), ScvO_2 _(76.5 ± 6.4% vs. 78.9 ± 5.8%) and serum lactate (*P *< 0.001) between patients with and without complications. After multivariate analysis, PCO_2 _gap and lactate level, but not ScvO_2_, were associated with postoperative complications (*P *< 0.001 and *P *= 0.018, respectively). Areas under the ROC curves were 0.66 (95% CI = 0.55 to 0.76) for lactate, 0.57 (95% CI = 0.46 to 0.68) for ScvO_2 _and 0.85 (95% CI = 0.77 to 0.93) for PCO_2 _gap, with 6 mmHg as the best threshold value for discriminating patients with and without complications. Patients with a PCO_2 _gap >6 mmHg (68%) had a longer duration of mechanical ventilation (4.1 ± 3.4 days vs. 5.6 ± 3.8 days, *P *= 0.047), and a longer hospital stay. Patients who died all had an enlarged PCO_2 _gap (*P *= 0.056).

## Conclusions

Both low and supranormal values of ScvO_2 _were found to be warning signals of impaired tissue oxygenation. A PCO_2 _gap larger than 6 mmHg could be a useful prognostic factor to identify patients at risk of postoperative complications following major abdominal surgery, especially when ScvO_2 _exceeds 75%.

